# Intensity of Predeath Grief and Postdeath Grief of Family Caregivers in Palliative Care in Relation to Preparedness for Caregiving, Caregiver Burden, and Social Support

**DOI:** 10.1089/pmr.2020.0033

**Published:** 2020-09-09

**Authors:** Lena Axelsson, Anette Alvariza, Maja Holm, Kristofer Årestedt

**Affiliations:** ^1^ Department of Nursing Science, Sophiahemmet University, Stockholm, Sweden.; ^2^ Department of Health Care Sciences/Palliative Research Centre, Ersta Sköndal Bräcke University College, Stockholm, Sweden.; ^3^ Capio Palliative Care, Dalen Hospital, Stockholm, Sweden.; ^4^ Faculty of Health and Life Sciences, Linnaeus University, Kalmar, Sweden.; ^5^ The Research Section, Region Kalmar County, Kalmar, Sweden.

**Keywords:** burden, family caregiver, grief, moderation, palliative care, preparedness

## Abstract

***Background:*** The intensity of predeath grief is associated with postdeath grief in family caregivers of patients in palliative care. Different factors during caregiving may influence this association.

***Objective:*** To examine (1) the intensity of grief in relation to preparedness for caregiving, caregiver burden, and social support, and (2) if these variables moderate associations between predeath and postdeath grief.

***Methods:*** This prospective correlational study used unpaired *t*-test to compare grief in relation to preparedness for caregiving, caregiver burden, and social support. Hierarchical multiple linear regression analysis investigated moderation effects. Family caregivers were recruited from 10 palliative homecare facilities. The Anticipatory Grief Scale, Texas Revised Inventory of Grief, Preparedness for Caregiving Scale, Caregiver Burden Scale, and Multidimensional Scale of Perceived Social Support were used. Ethical approval for the study was granted by the Regional Ethical Review Board in Stockholm, Sweden.

***Results:*** In total, 128 family caregivers participated. Those with high caregiver burden scored significantly higher intensity of predeath but not postdeath grief. Caregiver burden and social support moderated the association between intensity of predeath grief and postdeath grief. There was a stronger association between predeath and postdeath grief among caregivers with low caregiver burden or low social support. Preparedness for caregiving had no moderating effect.

***Discussion:*** Attention should be directed to caregiver burden and social support during family caregiving, as these variables seem to be significant for the intensity of grief before and after the patient's death. Acknowledging predeath grief during caregiving and recognizing pre- and postdeath grief as parts of the same process are of importance in clinical practice and when designing supportive interventions.

## Background

Family caregivers in a palliative care context face a demanding situation with physical and emotional strain and stress.^1,2^ The caregiver burden is often extensive^1,3^ with various health consequences^3–7^ and decreased quality of life.^3^ It is known that family caregivers who feel more prepared for the demands of the caregiver role tend to have more positive experiences of the caregiving situation.^8–10^ The caregiver burden can also be relieved through adequate social support, as it is found to be protective in stressful life events.^11,12^ In contrast, social isolation is found to be a risk factor in the caregiving situation.^3,13^

The situation of family caregivers is multifaceted and entails various losses, such as the relationship they had previously with the ill person, familiar daily life, and shared plans for the future. They often experience grief before the death of the ill person,^14^ which has earlier been suggested to alleviate bereavement after death.^15^ However, it has since been found that predeath grief does not reduce the grief work of bereavement.^16^ It is instead proposed that predeath and postdeath grief are parts of the same grief process.^17^

Predeath grief has recently been found to be associated with postdeath grief in family caregivers in palliative care.^16,17^ This association is complex and probably influenced by other factors in the caregiving situation. The *Integrative risk factor framework* for bereavement outcome,^18^ which is based on grief theories, describes bereavement as a process involving oscillation between focusing the loss itself and the new changes and possibilities in the continued life.^19,20^

The framework also states that interpersonal/contextual and intrapersonal factors may interplay in the individual grief process.^18^ It is correspondingly known, for example, that grief is associated with caregiver burden, preparedness, communication,^21^ anxiety, and depression.^22,23^ However, few studies have examined factors that may moderate the association between predeath grief and postdeath grief. By examining the role of moderation factors, the understanding of the grief process can be enhanced and patterns might be elucidated.

A previous study examined if symptoms of anxiety and depression could moderate the association between pre- and postdeath grief. Although symptoms of anxiety and depression were both associated with pre- and postdeath grief, no moderation effect on the association was shown.^17^ However, since there are many other factors during the complex caregiver period that may influence this association, this needs to be further investigated.

### Aim

The aim of this study was to examine (1) the intensity of grief in relation to preparedness for caregiving, caregiver burden, and social support, and (2) if these variables moderate the association between predeath grief and postdeath grief.

## Methods

### Study context

This study has a prospective correlational design using secondary analysis on data from a randomized intervention study^24^ in caregivers of patients with advanced incurable illness in specialized palliative home care. Ten facilities in a regional metropolitan catchment area that each provided palliative home care to between 70 and 200 patients were involved in the intervention study. Palliative care, including advanced symptom management and psychological support, was mainly performed by nurses in the patients' homes. Other professionals working at these facilities included physicians, occupational and physical therapists, social workers, and nutritionists. The psychoeducational intervention aimed to improve family caregivers' feelings of preparedness. It consisted of three group sessions focusing on physical, emotional, and existential issues related to the caregiving situation.

### Data collection

Family caregivers completed self-reported questionnaires at four time points: baseline, upon completion of the intervention, two months after the intervention, and six months after the patient's death. The baseline and postdeath measurements were used in this study. The baseline questionnaires, together with prepaid return envelopes, were distributed to the participants by health care professionals. Questionnaires for follow-up after the patient's death were sent by mail. The time span between baseline and the patient's death varied between seven months and two years.

### Measurements

This study used results from five validated self-reported instruments: The Anticipatory Grief Scale (AGS-13), Preparedness for Caregiving Scale (PCS), Caregiver Burden Scale (CBS), Multidimensional Scale of Perceived Social Support (MSPSS), and the Texas Revised Inventory of Grief (TRIG).

The AGS-13 is based on the original AGS, which was developed by Theut et al.^25^ and measures predeath grief in family caregivers. The original AGS, which is based partly on TRIG, consists of 27 items. All items are rated on a five-point Likert scale with a total score ranging from 27 to 135. Higher scores imply higher intensity of grief. In a validation study in the context of palliative care by Holm et al.,^26^ a 13-item version with two subscales was suggested: Behavioral reactions (eight items, possible score range from 8 to 40) and Emotional reactions (five items, possible score range from 5 to 25). In this study, both scales of the AGS-13 were used.

The PCS measures caregivers' perceived preparedness to provide care. The instrument was originally developed for the care of older persons in their own homes^27^ but has also demonstrated good measurement properties in the context of palliative care.^28,29^ The PCS consists of eight items rated on a five-point Likert-type scale. The responses are summed into a total score with a possible range between 0 and 32, where a higher score indicates higher levels of preparedness. Based on the median value in this study, the PCS was dichotomized into low (0–18) and high (19–32) preparedness to provide care.

The CBS measures self-perceived burden of caregivers and was developed in the context of care of persons with stroke.^30^ The instrument consists of 22 items divided into five dimensions: general strain, isolation, disappointment, emotional involvement, and environment. All items are rated on a four-point Likert-type scale (1–4). The scale scores are the ratio of the sum score of the responses in each domain divided by the number of items. The possible range of the scale scores is, therefore, between 1 and 4, where higher scores indicate a higher burden.^30^ In this study, only the general strain dimension (CBS-GS) was used. The scale was dichotomized into low (1.0–2.4) and high (2.5–4.0) caregiver burden based on the median value in this study.

The MSPSS measures perceived social support.^31^ The instrument is based on 12 items all rated on a seven-point Likert scale. The total score ranges between 12 and 84, where a higher score indicates a higher level of social support. In addition, three subscale scores can be calculated: family, friends, and significant others. Each subscale includes four items and has a possible score range between 4 and 28. The Swedish version has demonstrated good measurement properties.^32^ Only the total scale was used in this study and the scores were dichotomized into low (12–68) and high (69–84) social support based on the median value in this study.

The TRIG measures the intensity of grief after the death of a close person and is considered to cover normal grief processes.^33^ The instrument consists of 21 items, all rated on a five-point Likert scale. Lower scores imply higher levels of grief. Different subscales have been suggested in previous validation studies of TRIG. In a validation study by Holm et al.^34^ based on family caregivers in palliative care, two subscale scores were suggested: TRIG past feelings (8 items, possible score range 8–40) and TRIG present behaviors (13 items, possible score range 13–65). Both scales demonstrated good measurement properties in the context of palliative care. In this study, only TRIG present behaviors was used since the current situation concerning grief was the focus.

### Analysis

The intervention and control groups were analyzed as one sample since there were no significant differences in any of the study variables between the groups at baseline (AGS Behavioral, *p* = 0.740; AGS Emotional, *p* = 0.508; PCS, *p* = 0.384; CBS, *p* = 0.778; MSPSS, *p* = 0.379) or at follow-up (TRIG, *p* = 0.997).

Descriptive statistics were used to present baseline characteristics of family caregivers and study variables, including frequencies, means, and standard deviations.

Unpaired *t*-test was used to compare the intensity of grief in relation to persons with high versus low preparedness for caregiving, caregiver burden, and social support.

**FIG. 1.**
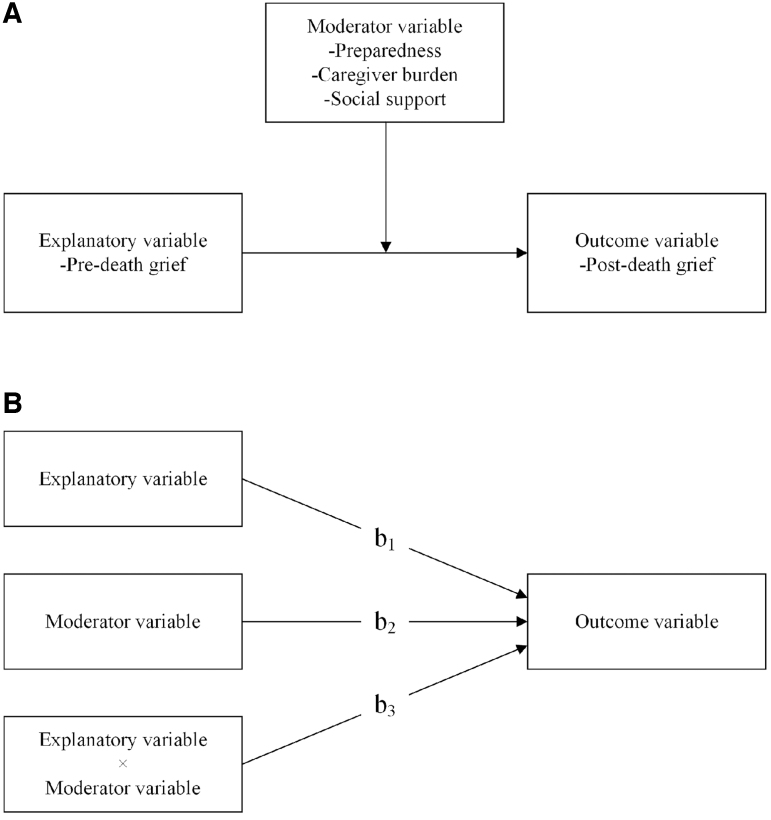
**(A)** The principles of moderation, that is, that the association between the explanatory variable and the outcome variable is affected by the moderator variable. **(B)** The regression model for a moderation analysis. Block I includes the explanatory variable (b1), Block II adds the moderator variable (b2), and Block III adds the multiplicative interaction effect (b3). b (b1–b3) represents the slope regression coefficient and a significant b3 suggests moderation.

Hierarchical multiple linear regression analyses were conducted to examine if preparedness for caregiving, caregiver burden, and social support, reported at baseline, moderated the association between predeath grief and postdeath grief. Moderation refers to examinations of the statistical interaction between one explanatory variable and one moderator variable predicting an outcome variable,^35^ as illustrated in Figure 1A. In total, six regression models were examined with predeath grief as the explanatory variable (AGS Behavioral reactions and AGS Emotional reactions) and postdeath grief (TRIG) as the outcome variable. Preparedness for caregiving (PCS), caregiver burden (CBS), and social support (MSPSS) were included as categorical moderator variables.

In accordance with Jose,^35^ each regression analysis was conducted in three blocks, as illustrated in Figure 1B. In block I (baseline model), postdeath grief was regressed on predeath grief. This step presents the main effect of predeath grief on postdeath grief. In block II (main effect model), the moderator variable was added to the model. The moderator variables were dummy coded with high preparedness, low caregiver burden, and high social support as reference categories, respectively (coded as 0). This step presents the main effect of predeath grief and the moderator variable on postdeath grief adjusted for each other. In block III (interaction effect model), a multiplicative interaction term between predeath grief and the moderator variable was added to the model (predeath grief × moderator variable). A significant interaction term implies that the association between the explanatory variable (predeath grief) and the outcome variable (postdeath grief) varies depending on the moderator variable. The main effect model and the interaction effect model were compared using the likelihood-ratio (LR) test. A significant LR test implies evidence of an interaction effect, that is, the association between predeath grief and postdeath grief is moderated by preparedness for caregiving, caregiver burden, and/or social support. Finally, identified interaction effects/moderations were illustrated by marginal mean plots.

The level of statistical significance was set at *p* < 0.05. Data were analyzed with Stata version 16.1 (StataCorp LP, College Station, TX, USA).

#### Ethical approval

Ethical approval for the study was granted by the Regional Ethical Review Board in Stockholm, Sweden (2012/377-31, 2012/2191-32, 2013/934-32).

## Results

### Family caregiver characteristics

Out of the 194 family caregivers at baseline, a total of 128 completed both baseline and postdeath measurements and were included in this study. They had a mean age of 62.0 (standard deviation = 13.2) years. Most were women (*n* = 85, 66%) and were either a spouse (*n* = 58, 45%) or an adult child (*n* = 44, 34%) of the patient. Nearly half (*n* = 61, 48%) were working (Table 1). Most of the patients (90%) had a cancer diagnosis.

**Table 1.** Family Caregiver Characteristics (*n* = 128)

Age, mean (SD)	62.0 (13.2)
Gender, *n* (%)	
Women	85 (66.4)
Men	43 (33.6)
Relation to patient, *n* (%)	
Spouse	58 (45.3)
Adult child	44 (34.4)
Other	26 (20.3)
Social status, *n* (%)	
Married/partner	90 (70.3)
Unmarried	38 (29.7)
Education level, *n* (%)	
Academic degree	56 (43.8)
Nonacademic degree	72 (56.2)
Occupation, *n* (%)	
Working	61 (47.7)
Retired	57 (44.5)
Other	10 (7.8)
Continuous study variables, mean (SD)	
Postdeath grief^a^	36.6 (11.8)
Predeath grief—Behavioral reactions^b^	19.4 (5.9)
Predeath grief—Emotional reactions^c^	14.6 (5.0)
Moderator variables	
Preparedness for caregiving, mean (SD)^d^	17.3 (6.9)
Dichotomized scores, *n* (%)	
Low preparedness (0–18)	72 (56.3)
High preparedness (19–32)	56 (43.8)
Caregiver burden—general strain, mean (SD)^e^	2.3 (0.7)
Dichotomized scores, *n* (%)	
Low caregiver burden (1.0–2.4)	73 (57.0)
High caregiver burden (2.5–4.0)	55 (43.0)
Social support, mean (SD)^f^	64.8 (15.8)
Dichotomized scores, *n* (%)	
Low social support (12–68)	67 (52.3)
High social support (69–84)	61 (47.7)

^a^ Texas Revised Inventory of Grief, possible score range 13–65, measured postdeath, not at baseline.

^b^ Anticipatory Grief Scale—Behavioral reactions, possible score range 8–40.

^c^ Anticipatory Grief Scale—Emotional reactions, possible score range 5–25.

^d^ Preparedness for Caregiving Scale, possible score range 0–32.

^e^ Caregiver Burden Scale—general strain, possible score range 1–4.

^f^ Multidimensional Scale of Perceived Social Support, possible score range 12–84.

SD, standard deviation.

### The intensity of predeath grief and postdeath grief in relation to preparedness for caregiving, caregiver burden, and/or social support

Family caregivers with high caregiver burden scored significantly higher intensity of predeath grief for both Behavioral (*p* < 0.001) and Emotional (*p* = 0.001) reactions. In contrast, there were no differences in reported predeath grief between family caregivers with high or low preparedness for caregiving or social support (Table 2). Furthermore, there were no significant differences in reported postdeath grief between family caregivers with high or low preparedness for caregiving, caregiver burden, and/or social support (Table 2).

**Table 2.** Intensity of Grief in Relation to Preparedness for Caregiving, Caregiver Burden, and Social Support

	Predeath grief					
	Behavioral reactions		Emotional reactions		Postdeath grief	
Moderator variables	Mean (SD)	p^a^	Mean (SD)	p^a^	Mean (SD)	p^a^
Preparedness						
Low	20.2 (6.1)	0.084	14.8 (4.9)	0.558	36.6 (12.1)	0.958
High	18.4 (5.5)		14.3 (5.2)		36.7 (11.6)	
Caregiver burden						
Low	16.6 (4.3)	<0.001	13.4 (5.1)	0.001	38.3 (12.6)	0.068
High	23.1(5.7)		16.2 (4.4)		34.4 (10.5)	
Social support						
Low	20.3 (5.8)	0.070	14.5 (5.1)	0.796	35.0 (12.4)	0.102
High	18.4 (5.9)		14.7 (4.9)		38.4 (11.0)	

^a^ Unpaired sample *t*-test.

### Moderation effects of preparedness for caregiving, caregiver burden, and social support on the association between intensity of predeath grief and postdeath grief

The baseline models showed significant associations between intensity of predeath grief and postdeath grief. Higher predeath grief was associated with higher postdeath grief for both Behavioral reactions (*B* = −0.91, *p* < 0.001) and Emotional reactions (*B* = −1.17, *p* < 0.001). Behavioral reactions and Emotional reactions in predeath grief explained 20% and 24%, respectively, of the total variance in postdeath grief (Tables 3 and 4).

**Table 3.** Associations between Intensity of Predeath Grief (Behavioral Reactions) and Postdeath Grief, Including Interaction Effects of Preparedness for Caregiving, Caregiver Burden General Strain, and Social Support (*n* = 128)

Explanatory variables	Block I: Baseline model		Block II: Main effect model		Block III: Interaction effect model	
	b	p	b	p	b	p
Predeath grief—Behavioral reactions	−0.91	<0.001	−0.93	<0.001	−0.94	<0.001
Preparedness for caregiving			1.57	0.414	1.27	0.849
Predeath grief × preparedness					0.02	0.963
Model statistics	*F*(1, 126) = 32.3, *p* < 0.001, *R*^2^ = 0.20		*F*(2, 125) = 16.4, *p* < 0.001, *R*^2^ = 0.21		*F*(3, 124) = 10.9, *p* < 0.001, *R*^2^ = 0.21	
Predeath grief—Behavioral reactions	−0.91	<0.001	−1.04	<0.001	−1.63	<0.001
Caregiver burden—General strain			2.90	0.200	−17.35	0.024
Predeath grief × caregiver burden					1.04	0.006
Model statistics	*F*(1, 126) = 32.3, *p* < 0.001, *R*^2^ = 0.20		*F*(2, 125) = 17.1, *p* < 0.001, *R*^2^ = 0.21		*F*(3, 124) = 14.6, *p* < 0.001, *R*^2^ = 0.26	
Predeath grief—Behavioral reactions	−0.91	<0.001	−0.88	<0.001	−0.70	0.003
Social support			−1.76	0.356	5.08	0.439
Behavioral reactions × social support					−0.35	0.277
Model statistics	*F*(1, 126) = 32.3, *p* < 0.001, *R*^2^ = 0.20		*F*(2, 125) = 16.6, *p* < 0.001, *R*^2^ = 0.21		*F*(3, 124) = 11.5, *p* < 0.001, *R*^2^ = 0.22	

**Table 4.** Associations between Intensity of Predeath Grief (Emotional Reactions) and Postdeath Grief, Including Interaction Effects of Preparedness for Caregiving, Caregiver Burden General Strain, and Social Support (*n* = 128)

Explanatory variables	Block I:Baseline model		Block II:Main effect model		Block III: Interaction effect model	
	b	p	b	p	b	p
Predeath grief—Emotional reactions	−1.17	<0.001	−1.17	<0.001	−1.15	<0.001
Preparedness for caregiving			0.50	0.787	0.89	0.875
Predeath grief × preparedness					−0.03	0.942
Model statistics	*F*(1, 126) = 40.6, *p* < 0.001, *R*^2^ = 0.24		*F*(2, 125) = 20.2, *p* < 0.001, *R*^2^ = 0.24		*F*(3, 124) = 13.4, *p* < 0.001, *R*^2^ = 0.24	
Predeath grief—Emotional reactions	−1.17	<0.001	−1.15	<0.001	−1.44	<0.001
Caregiver burden—General strain			−0.59	0.760	−12.91	0.042
Predeath grief × caregiver burden					0.81	0.042
Model statistics	*F*(1, 126) = 40.6, *p* < 0.001, *R*^2^ = 0.24		*F*(2, 125) = 20.2, *p* < 0.001, *R*^2^ = 0.24		*F*(3, 124) = 15.2, *p* < 0.001, *R*^2^ = 0.27	
Predeath grief—emotional reactions	−1.17	<0.001	−1.17	<0.001	−0.72	0.007
Social support			−3.70	0.043	8.61	0.120
Predeath grief × social support					−0.84	0.020
Model statistics	*F*(1, 126) = 40.6, *p* < 0.001, *R*^2^ = 0.24		*F*(2, 125) = 22.9, *p* < 0.001, *R*^2^ = 0.27		*F*(3, 124) = 17.7, *p* < 0.001, *R*^2^ = 0.30	

In the main effect models, the significant associations between intensity of predeath grief and postdeath grief also persisted when preparedness for caregiving, caregiver burden, or social support were added to the model (Tables 3 and 4).

**FIG. 2.**
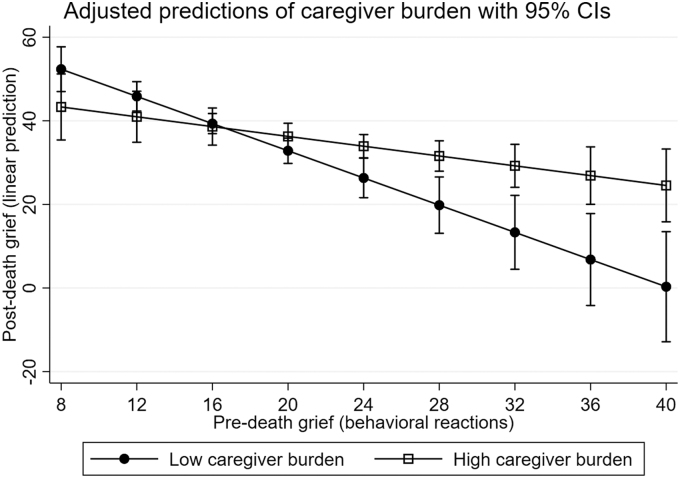
Marginal mean plot of the association between the intensity of predeath (Behavioral reactions) and postdeath grief by caregiver burden. A stronger association between predeath and postdeath grief is shown for family caregivers who reported low caregiver burden compared with those who reported high caregiver burden.

**FIG. 3.**
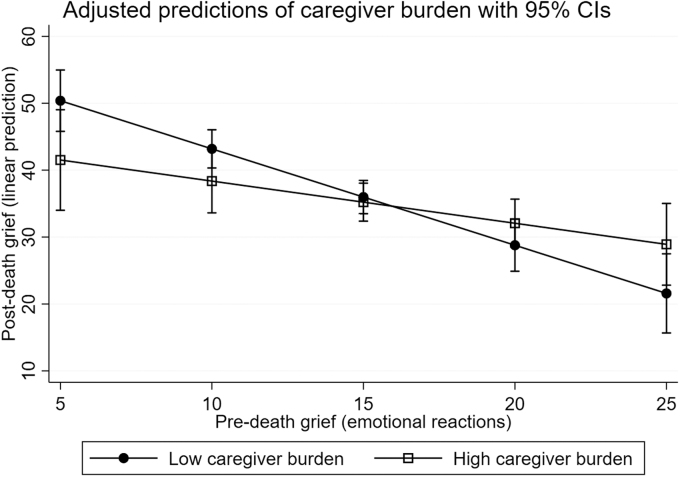
Marginal mean plot of the association between the intensity of predeath (Emotional reactions) and postdeath grief by caregiver burden. A stronger association between predeath and postdeath grief is shown for family caregivers who reported low caregiver burden compared with those who reported high caregiver burden.

The interaction effect models showed that preparedness for caregiving did not moderate the association between the intensity of predeath grief and postdeath grief. However, both caregiver burden and social support moderated the association between predeath grief and postdeath grief (Tables 3 and 4). Caregiver burden moderated the association between predeath grief and postdeath grief for both Behavioral reactions (*B* = 1.04, *p* = 0.006) (Table 3) and Emotional reactions (*B* = 0.81, *p* = 0.042) (Table 4). These moderations were confirmed by the LR test [*χ*^2^(1) = 7.75, *p* = 0.005 vs. *χ*^2^(1) = 4.30, *p* = 0.038, respectively]. The moderations are illustrated in Figure 2 (Behavioral reactions) and Figure 3 (Emotional reactions). The illustrations show that the association between intensity of predeath grief and postdeath grief was significantly stronger among caregivers who reported low caregiver burden compared with those reporting high caregiver burden.

**FIG. 4.**
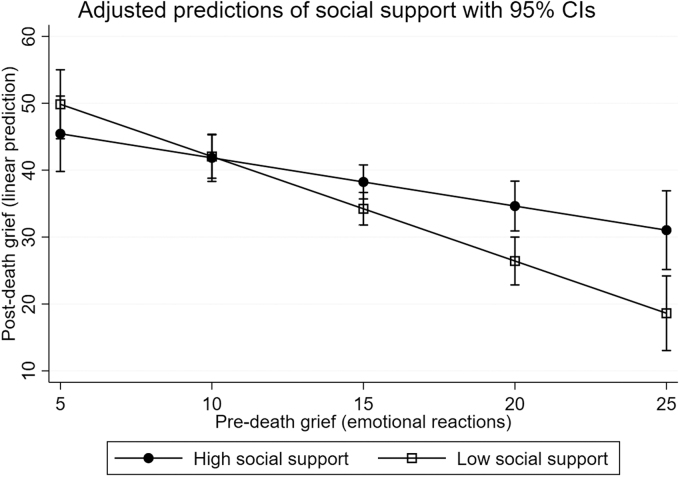
Marginal mean plot of the association between the intensity of predeath (Emotional reactions) and postdeath grief by social support. A stronger association between predeath and postdeath grief is shown for family caregivers who reported low social support compared with those who reported high social support.

Also social support moderated the association between predeath grief and postdeath grief, but only for predeath grief Emotional reactions (*B* = −0.84, *p* = 0.020) (Table 4). This moderation was confirmed by the LR test [*χ*^2^(1) = 5.64, *p* = 0.018]. The association between intensity of predeath grief and postdeath grief was significantly stronger among caregivers who reported low social support compared with those reporting high social support, as illustrated in Figure 4.

## Discussion

This study showed that caregivers in palliative home care with a high caregiver burden scored significantly higher predeath grief. Moreover, the association between the intensity of predeath grief and postdeath grief was found to be moderated by caregiver burden and social support. Thus, there was a stronger association between predeath grief and postdeath grief among caregivers with low caregiver burden or low social support.

Since high caregiver burden was related to significantly higher intensity of predeath grief but not postdeath grief, this may reflect that experiences of the caregiver burden are critical for experiences of predeath losses and stress in the caregiving situation,^1,14^ emphasizing a need for caregiver support predeath. These results are also in line with findings that severe symptoms of predeath grief in caregivers of patients with cancer at the end of life were associated with a high caregiver burden^21^ and that family caregivers experienced higher levels of grief symptoms during caregiving compared with six months after the patient's death.^23^ It is also found that a demanding period of suffering and burden before death may be experienced as being worse than bereavement and family caregivers may even feel relieved after the death of the patient.^14,36^

However, our results of moderation effects between variables show that the association between the intensity of predeath grief and postdeath grief was significantly stronger among caregivers who reported low caregiver burden compared with those reporting high caregiver burden. Thus, a family caregiver may experience intense predeath grief and postdeath grief despite a low caregiver burden. This pattern increases the understanding of the complexity of the grief process, which shows the importance of assessing the family caregivers' situation and individualizing the provision and design of support predeath and postdeath.

Although our findings showed no differences in pre- or postdeath grief in relation to social support, we found that social support moderated the association between predeath grief and postdeath grief. This association was significantly stronger among caregivers who reported low social support, which suggests that caregivers who reported high predeath grief together with low social support also reported high postdeath grief. The significance of social support for bereaved family caregivers has also been demonstrated in earlier research. For example, social support has been found to be correlated with the intensity of grief, and also with complicated grief in family caregivers.^37^ Family caregivers have described feelings of social isolation in the caregiving situation and the need for gaining strength and support from family or friends in the form of communication or respite.^2,38,39^ Altogether this suggests the importance of enhanced social support from family, friends, or significant others during caregiving.

There were no differences in pre- or postdeath grief between family caregivers with high or low preparedness for caregiving, and preparedness for caregiving did not moderate the association between intensity of predeath grief and postdeath grief. These results might be unexpected since family caregivers in earlier studies have described preparedness for caregiving and preparedness for death as closely related to each other. Grief and thoughts about death were always present during the caregiving period.^40,41^ Feeling prepared for an expected death has also been described as beneficial in bereavement^42,43^ and low preparedness for the relative's death is found to be associated with predeath grief symptoms.^21^

However, preparedness for caregiving and feeling prepared for an expected death are different concepts with different measures, which may explain this study results. Nevertheless, since preparedness for caregiving is associated with experiences of the caregiving situation^8,44,45^ it is reasonable to think that preparedness still influences other variables of significance, such as caregiver burden and, therefore, has a role in the interaction of variables in the complex grief process.

### Limitations

There are some limitations to this study that should be considered. One of the limitations is that the moderator variables were dichotomized using the median value. The reason for this is that the PCS, CBS, and MSPSS do not have an established cutoff score. Another limitation is that predeath grief and postdeath grief were measured with different instruments, which may influence the results. However, the original AGS is partly based on TRIG and both instruments are validated in the palliative care context.^26,34^

Moreover, although predeath grief and postdeath grief are related, they are not the same constructs and, to the best of our knowledge, there are no instruments developed that can measure both aspects of normal grief. The moderator variables were measured at baseline, when predeath grief was also measured, which means that changes over time are not considered. However, results still show the impact of predeath caregiver burden and social support on family caregivers' grief. Finally, no *a priori* power analysis was conducted specifically for this study since it is part of a larger intervention study. However, the sample can be considered large enough since a regression model, including three explanatory variables, requires at least 77 observations to detect a medium effect size (*f*^2^ = 0.15, *α* = 0.05, 1 − *β* = 0.80).

## Conclusions

The results of this prospective correlational study in a palliative care context add to knowledge about the complexity and significance of caregiver burden and social support in relation to the grief process of family caregivers. The results demonstrate that a high caregiver burden is associated with higher predeath grief. Furthermore, results show that the association between intensity of predeath grief and postdeath grief in family caregivers could be moderated, especially by low caregiver burden and low social support. Hence, the results emphasize that special attention should be directed to these variables, bearing in mind that, together with high predeath grief, low caregiver burden may also imply a need for support in bereavement.

Acknowledging predeath grief during caregiving and recognizing predeath grief and postdeath grief as parts of the same process are of importance, both in everyday clinical practice and when designing supportive interventions. Family caregivers may benefit from conversations with health care professionals, including opportunities to talk about their individual resources and needs.^46–49^ However, further research is needed to enhance the understanding of the interplay between different variables in the grief process. Factors of interest are, for example, the significance of mutuality between the patient and family caregiver as well as relationship quality.

## Abbreviations Used

AGS-13: The Anticipatory Grief Scale
CBS: Caregiver Burden Scale
LR: likelihood-ratio
MSPSS: Multidimensional Scale of Perceived Social Support
PCS: Preparedness for Caregiving Scale
SD: standard deviation
TRIG: Texas Revised Inventory of Grief
